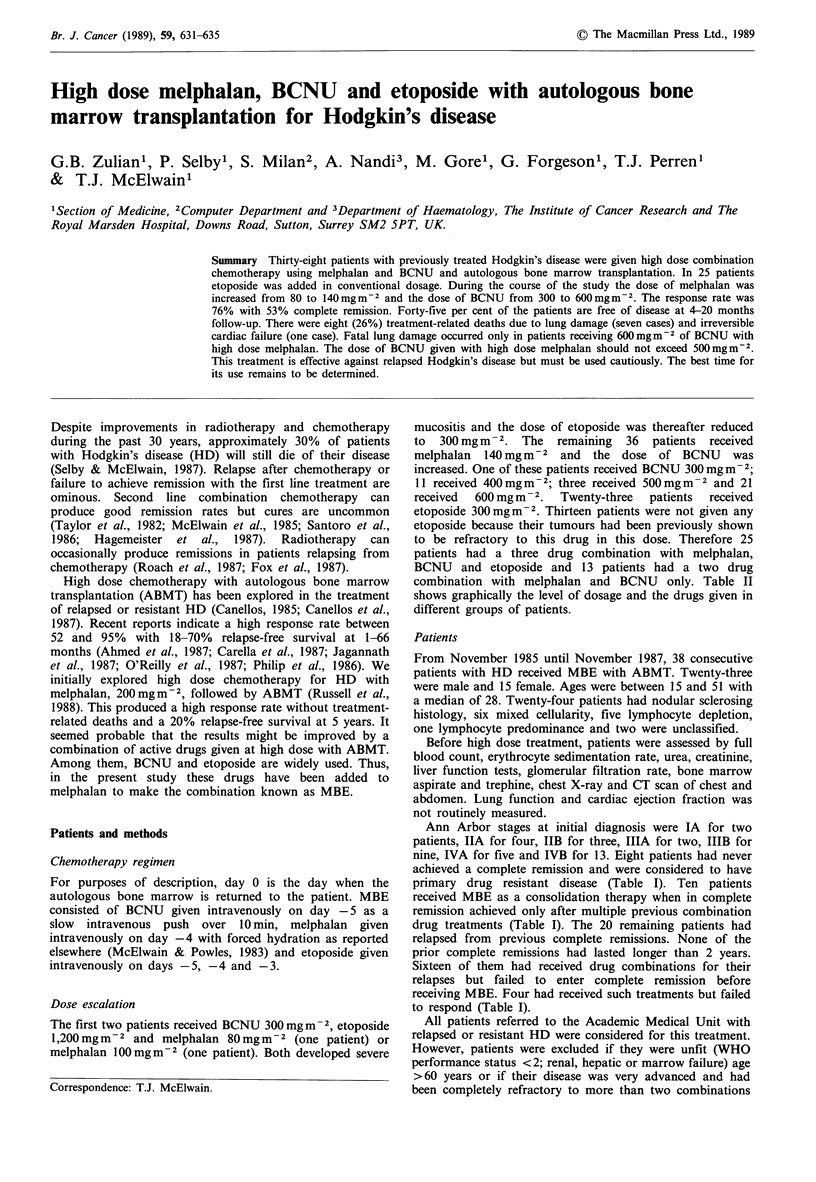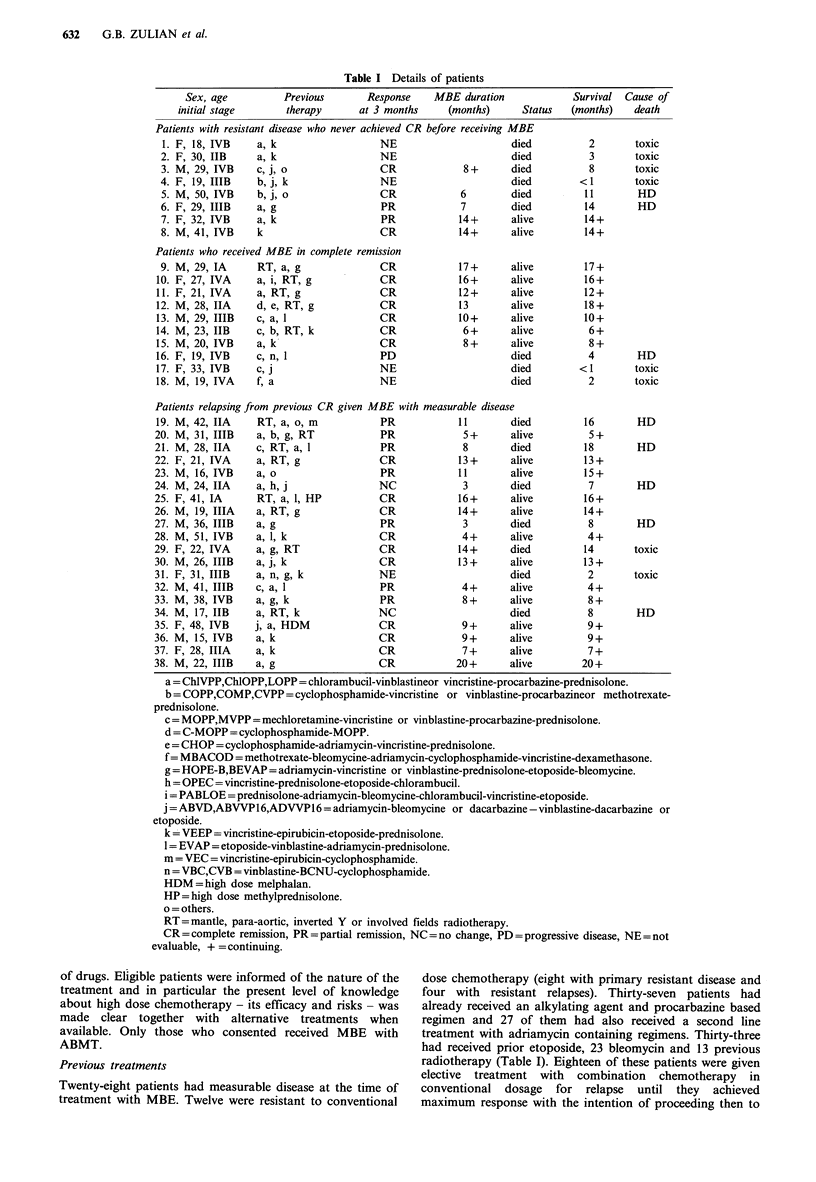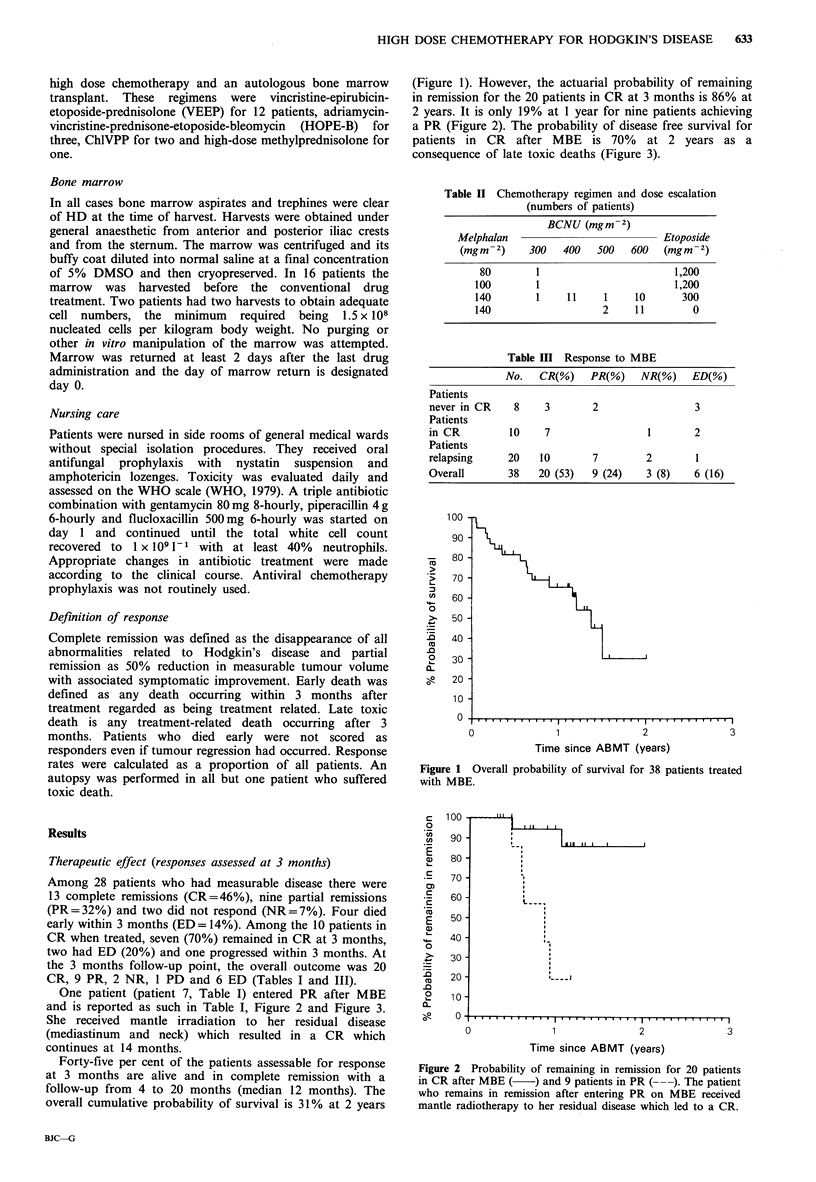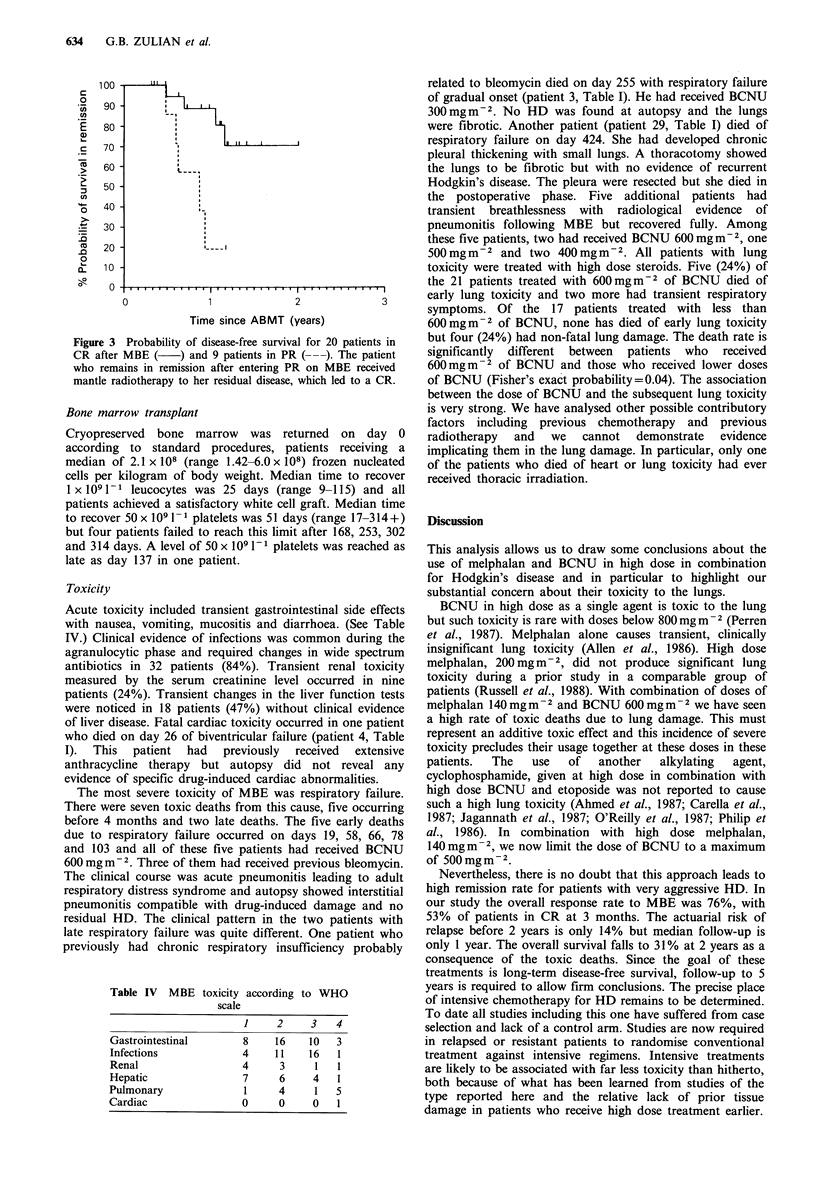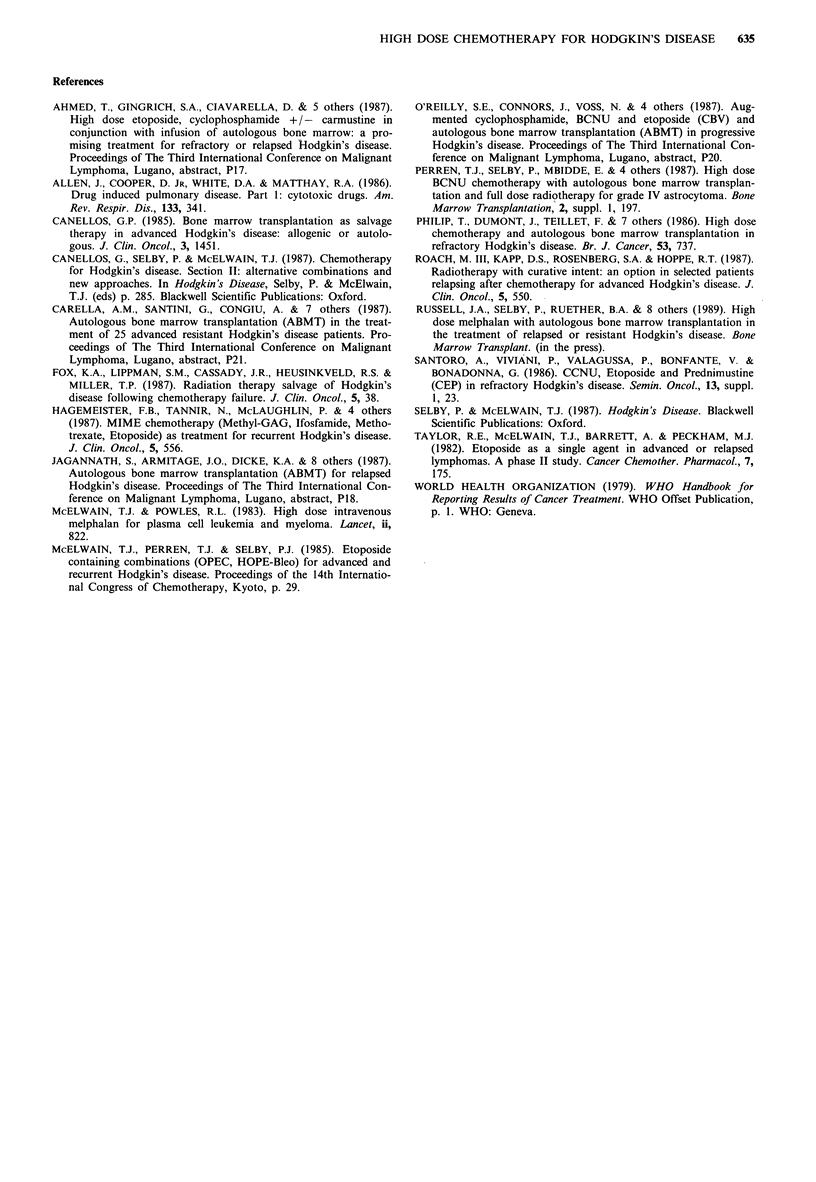# High dose melphalan, BCNU and etoposide with autologous bone marrow transplantation for Hodgkin's disease.

**DOI:** 10.1038/bjc.1989.128

**Published:** 1989-04

**Authors:** G. B. Zulian, P. Selby, S. Milan, A. Nandi, M. Gore, G. Forgeson, T. J. Perren, T. J. McElwain

**Affiliations:** Section of Medicine, Royal Marsden Hospital, Sutton, Surrey, UK.

## Abstract

Thirty-eight patients with previously treated Hodgkin's disease were given high dose combination chemotherapy using melphalan and BCNU and autologous bone marrow transplantation. In 25 patients etoposide was added in conventional dosage. During the course of the study the dose of melphalan was increased from 80 to 140 mg m-2 and the dose of BCNU from 300 to 600 mg m-2. The response rate was 76% with 53% complete remission. Forty-five per cent of the patients are free of disease at 4-20 months follow-up. There were eight (26%) treatment-related deaths due to lung damage (seven cases) and irreversible cardiac failure (one case). Fatal lung damage occurred only in patients receiving 600 mg m-2 of BCNU with high dose melphalan. The dose of BCNU given with high dose melphalan should not exceed 500 mg m-2. This treatment is effective against relapsed Hodgkin's disease but must be used cautiously. The best time for its use remains to be determined.


Br. J. Cancer (1989), 59, 631–635 © The Macmillan Press Ltd., 1989

### High dose melphalan, BCNU and etoposide with autologous bone marrow transplantation for Hodgkin's disease

G.B. Zulian[1], P. Selby[1], S. Milan[2], A. Nandi[3], M. Gore[1], G. Forgeson[1], T.J. Perren[1] & T.J. McElwain[1]

[1]*Section of Medicine,* [2]*Computer Department and* [3]*Department of Haematology, The Institute of Cancer Research and The Royal Marsden Hospital, Downs Road, Sutton, Surrey SM2 5PT, UK.*

**Summary** Thirty-eight patients with previously treated Hodgkin's disease were given high dose combination chemotherapy using melphalan and BCNU and autologous bone marrow transplantation. In 25 patients etoposide was added in conventional dosage. During the course of the study the dose of melphalan was increased from 80 to 140 mg m$^{-2}$ and the dose of BCNU from 300 to 600 mg m$^{-2}$. The response rate was 76% with 53% complete remission. Forty-five per cent of the patients are free of disease at 4–20 months follow-up. There were eight (26%) treatment-related deaths due to lung damage (seven cases) and irreversible cardiac failure (one case). Fatal lung damage occurred only in patients receiving 600 mg m$^{-2}$ of BCNU with high dose melphalan. The dose of BCNU given with high dose melphalan should not exceed 500 mg m$^{-2}$. This treatment is effective against relapsed Hodgkin's disease but must be used cautiously. The best time for its use remains to be determined.

Despite improvements in radiotherapy and chemotherapy during the past 30 years, approximately 30% of patients with Hodgkin's disease (HD) will still die of their disease (Selby & McElwain, 1987). Relapse after chemotherapy or failure to achieve remission with the first line treatment are ominous. Second line combination chemotherapy can produce good remission rates but cures are uncommon (Taylor *et al.*, 1982; McElwain *et al.*, 1985; Santoro *et al.*, 1986; Hagemeister *et al.*, 1987). Radiotherapy can occasionally produce remissions in patients relapsing from chemotherapy (Roach *et al.*, 1987; Fox *et al.*, 1987).

High dose chemotherapy with autologous bone marrow transplantation (ABMT) has been explored in the treatment of relapsed or resistant HD (Canellos, 1985; Canellos *et al.*, 1987). Recent reports indicate a high response rate between 52 and 95% with 18–70% relapse-free survival at 1–66 months (Ahmed *et al.*, 1987; Carella *et al.*, 1987; Jagannath *et al.*, 1987; O'Reilly *et al.*, 1987; Philip *et al.*, 1986). We initially explored high dose chemotherapy for HD with melphalan, 200 mg m$^{-2}$, followed by ABMT (Russell *et al.*, 1988). This produced a high response rate without treatment-related deaths and a 20% relapse-free survival at 5 years. It seemed probable that the results might be improved by a combination of active drugs given at high dose with ABMT. Among them, BCNU and etoposide are widely used. Thus, in the present study these drugs have been added to melphalan to make the combination known as MBE.

### Patients and methods

### *Chemotherapy regimen*

For purposes of description, day 0 is the day when the autologous bone marrow is returned to the patient. MBE consisted of BCNU given intravenously on day −5 as a slow intravenous push over 10 min, melphalan given intravenously on day −4 with forced hydration as reported elsewhere (McElwain & Powles, 1983) and etoposide given intravenously on days −5, −4 and −3.

### *Dose escalation*

The first two patients received BCNU 300 mg m$^{-2}$, etoposide 1,200 mg m$^{-2}$ and melphalan 80 mg m$^{-2}$ (one patient) or melphalan 100 mg m$^{-2}$ (one patient). Both developed severe mucositis and the dose of etoposide was thereafter reduced to 300 mg m$^{-2}$. The remaining 36 patients received melphalan 140 mg m$^{-2}$ and the dose of BCNU was increased. One of these patients received BCNU 300 mg m$^{-2}$; 11 received 400 mg m$^{-2}$; three received 500 mg m$^{-2}$ and 21 received 600 mg m$^{-2}$. Twenty-three patients received etoposide 300 mg m$^{-2}$. Thirteen patients were not given any etoposide because their tumours had been previously shown to be refractory to this drug in this dose. Therefore 25 patients had a three drug combination with melphalan, BCNU and etoposide and 13 patients had a two drug combination with melphalan and BCNU only. Table II shows graphically the level of dosage and the drugs given in different groups of patients.

### *Patients*

From November 1985 until November 1987, 38 consecutive patients with HD received MBE with ABMT. Twenty-three were male and 15 female. Ages were between 15 and 51 with a median of 28. Twenty-four patients had nodular sclerosing histology, six mixed cellularity, five lymphocyte depletion, one lymphocyte predominance and two were unclassified.

Before high dose treatment, patients were assessed by full blood count, erythrocyte sedimentation rate, urea, creatinine, liver function tests, glomerular filtration rate, bone marrow aspirate and trephine, chest X-ray and CT scan of chest and abdomen. Lung function and cardiac ejection fraction was not routinely measured.

Ann Arbor stages at initial diagnosis were IA for two patients, IIA for four, IIB for three, IIIA for two, IIIB for nine, IVA for five and IVB for 13. Eight patients had never achieved a complete remission and were considered to have primary drug resistant disease (Table I). Ten patients received MBE as a consolidation therapy when in complete remission achieved only after multiple previous combination drug treatments (Table I). The 20 remaining patients had relapsed from previous complete remissions. None of the prior complete remissions had lasted longer than 2 years. Sixteen of them had received drug combinations for their relapses but failed to enter complete remission before receiving MBE. Four had received such treatments but failed to respond (Table I).

All patients referred to the Academic Medical Unit with relapsed or resistant HD were considered for this treatment. However, patients were excluded if they were unfit (WHO performance status <2; renal, hepatic or marrow failure) age >60 years or if their disease was very advanced and had been completely refractory to more than two combinations

Correspondence: T.J. McElwain.

**Table I** Details of patients

| Sex, age initial stage | Previous therapy | Response at 3 months | MBE duration (months) | Status | Survival (months) | Cause of death |
|---|---|---|---|---|---|---|
| *Patients with resistant disease who never achieved CR before receiving MBE* | | | | | | |
| 1. F, 18, IVB | a, k | NE | | died | 2 | toxic |
| 2. F, 30, IIB | a, k | NE | | died | 3 | toxic |
| 3. M, 29, IVB | c, j, o | CR | 8+ | died | 8 | toxic |
| 4. F, 19, IIIB | b, j, k | NE | | died | <1 | toxic |
| 5. M, 50, IVB | b, j, o | CR | 6 | died | 11 | HD |
| 6. F, 29, IIIB | a, g | PR | 7 | died | 14 | HD |
| 7. F, 32, IVB | a, k | PR | 14+ | alive | 14+ | |
| 8. M, 41, IVB | k | CR | 14+ | alive | 14+ | |
| *Patients who received MBE in complete remission* | | | | | | |
| 9. M, 29, IA | RT, a, g | CR | 17+ | alive | 17+ | |
| 10. F, 27, IVA | a, i, RT, g | CR | 16+ | alive | 16+ | |
| 11. F, 21, IVA | a, RT, g | CR | 12+ | alive | 12+ | |
| 12. M, 28, IIA | d, e, RT, g | CR | 13 | alive | 18+ | |
| 13. M, 29, IIIB | c, a, l | CR | 10+ | alive | 10+ | |
| 14. M, 23, IIB | c, b, RT, k | CR | 6+ | alive | 6+ | |
| 15. M, 20, IVB | a, k | CR | 8+ | alive | 8+ | |
| 16. F, 19, IVB | c, n, l | PD | | died | 4 | HD |
| 17. F, 33, IVB | c, j | NE | | died | <1 | toxic |
| 18. M, 19, IVA | f, a | NE | | died | 2 | toxic |
| *Patients relapsing from previous CR given MBE with measurable disease* | | | | | | |
| 19. M, 42, IIA | RT, a, o, m | PR | 11 | died | 16 | HD |
| 20. M, 31, IIIB | a, b, g, RT | PR | 5+ | alive | 5+ | |
| 21. M, 28, IIA | c, RT, a, l | PR | 8 | died | 18 | HD |
| 22. F, 21, IVA | a, RT, g | CR | 13+ | alive | 13+ | |
| 23. M, 16, IVB | a, o | PR | 11 | alive | 15+ | |
| 24. M, 24, IIA | a, h, j | NC | 3 | died | 7 | HD |
| 25. F, 41, IA | RT, a, l, HP | CR | 16+ | alive | 16+ | |
| 26. M, 19, IIIA | a, RT, g | CR | 14+ | alive | 14+ | |
| 27. M, 36, IIIB | a, g | PR | 3 | died | 8 | HD |
| 28. M, 51, IVB | a, l, k | CR | 4+ | alive | 4+ | |
| 29. F, 22, IVA | a, g, RT | CR | 14+ | died | 14 | toxic |
| 30. M, 26, IIIB | a, j, k | CR | 13+ | alive | 13+ | |
| 31. F, 31, IIIB | a, n, g, k | NE | | died | 2 | toxic |
| 32. M, 41, IIIB | c, a, l | PR | 4+ | alive | 4+ | |
| 33. M, 38, IVB | a, g, k | PR | 8+ | alive | 8+ | |
| 34. M, 17, IIB | a, RT, k | NC | | died | 8 | HD |
| 35. F, 48, IVB | j, a, HDM | CR | 9+ | alive | 9+ | |
| 36. M, 15, IVB | a, k | CR | 9+ | alive | 9+ | |
| 37. F, 28, IIIA | a, k | CR | 7+ | alive | 7+ | |
| 38. M, 22, IIIB | a, g | CR | 20+ | alive | 20+ | |

a = ChlVPP,ChlOPP,LOPP = chlorambucil-vinblastineor vincristine-procarbazine-prednisolone.
b = COPP,COMP,CVPP = cyclophosphamide-vincristine or vinblastine-procarbazineor methotrexate-prednisolone.
c = MOPP,MVPP = mechloretamine-vincristine or vinblastine-procarbazine-prednisolone.
d = C-MOPP = cyclophosphamide-MOPP.
e = CHOP = cyclophosphamide-adriamycin-vincristine-prednisolone.
f = MBACOD = methotrexate-bleomycine-adriamycin-cyclophosphamide-vincristine-dexamethasone.
g = HOPE-B,BEVAP = adriamycin-vincristine or vinblastine-prednisolone-etoposide-bleomycine.
h = OPEC = vincristine-prednisolone-etoposide-chlorambucil.
i = PABLOE = prednisolone-adriamycin-bleomycine-chlorambucil-vincristine-etoposide.
j = ABVD,ABVVP16,ADVVP16 = adriamycin-bleomycine or dacarbazine – vinblastine-dacarbazine or etoposide.
k = VEEP = vincristine-epirubicin-etoposide-prednisolone.
l = EVAP = etoposide-vinblastine-adriamycin-prednisolone.
m = VEC = vincristine-epirubicin-cyclophosphamide.
n = VBC,CVB = vinblastine-BCNU-cyclophosphamide.
HDM = high dose melphalan.
HP = high dose methylprednisolone.
o = others.
RT = mantle, para-aortic, inverted Y or involved fields radiotherapy.
CR = complete remission, PR = partial remission, NC = no change, PD = progressive disease, NE = not evaluable, + = continuing.

of drugs. Eligible patients were informed of the nature of the treatment and in particular the present level of knowledge about high dose chemotherapy – its efficacy and risks – was made clear together with alternative treatments when available. Only those who consented received MBE with ABMT.

### *Previous treatments*

Twenty-eight patients had measurable disease at the time of treatment with MBE. Twelve were resistant to conventional dose chemotherapy (eight with primary resistant disease and four with resistant relapses). Thirty-seven patients had already received an alkylating agent and procarbazine based regimen and 27 of them had also received a second line treatment with adriamycin containing regimens. Thirty-three had received prior etoposide, 23 bleomycin and 13 previous radiotherapy (Table I). Eighteen of these patients were given elective treatment with combination chemotherapy in conventional dosage for relapse until they achieved maximum response with the intention of proceeding then to high dose chemotherapy and an autologous bone marrow transplant. These regimens were vincristine-epirubicin-etoposide-prednisolone (VEEP) for 12 patients, adriamycin-vincristine-prednisone-etoposide-bleomycin (HOPE-B) for three, ChlVPP for two and high-dose methylprednisolone for one.

*Bone marrow*

In all cases bone marrow aspirates and trephines were clear of HD at the time of harvest. Harvests were obtained under general anaesthetic from anterior and posterior iliac crests and from the sternum. The marrow was centrifuged and its buffy coat diluted into normal saline at a final concentration of 5% DMSO and then cryopreserved. In 16 patients the marrow was harvested before the conventional drug treatment. Two patients had two harvests to obtain adequate cell numbers, the minimum required being $1.5 \times 10^8$ nucleated cells per kilogram body weight. No purging or other *in vitro* manipulation of the marrow was attempted. Marrow was returned at least 2 days after the last drug administration and the day of marrow return is designated day 0.

*Nursing care*

Patients were nursed in side rooms of general medical wards without special isolation procedures. They received oral antifungal prophylaxis with nystatin suspension and amphotericin lozenges. Toxicity was evaluated daily and assessed on the WHO scale (WHO, 1979). A triple antibiotic combination with gentamycin 80 mg 8-hourly, piperacillin 4 g 6-hourly and flucloxacillin 500 mg 6-hourly was started on day 1 and continued until the total white cell count recovered to $1 \times 10^9 \, l^{-1}$ with at least 40% neutrophils. Appropriate changes in antibiotic treatment were made according to the clinical course. Antiviral chemotherapy prophylaxis was not routinely used.

*Definition of response*

Complete remission was defined as the disappearance of all abnormalities related to Hodgkin's disease and partial remission as 50% reduction in measurable tumour volume with associated symptomatic improvement. Early death was defined as any death occurring within 3 months after treatment regarded as being treatment related. Late toxic death is any treatment-related death occurring after 3 months. Patients who died early were not scored as responders even if tumour regression had occurred. Response rates were calculated as a proportion of all patients. An autopsy was performed in all but one patient who suffered toxic death.

### Results

*Therapeutic effect (responses assessed at 3 months)*

Among 28 patients who had measurable disease there were 13 complete remissions (CR = 46%), nine partial remissions (PR = 32%) and two did not respond (NR = 7%). Four died early within 3 months (ED = 14%). Among the 10 patients in CR when treated, seven (70%) remained in CR at 3 months, two had ED (20%) and one progressed within 3 months. At the 3 months follow-up point, the overall outcome was 20 CR, 9 PR, 2 NR, 1 PD and 6 ED (Tables I and III).

One patient (patient 7, Table I) entered PR after MBE and is reported as such in Table I, Figure 2 and Figure 3. She received mantle irradiation to her residual disease (mediastinum and neck) which resulted in a CR which continues at 14 months.

Forty-five per cent of the patients assessable for response at 3 months are alive and in complete remission with a follow-up from 4 to 20 months (median 12 months). The overall cumulative probability of survival is 31% at 2 years (Figure 1). However, the actuarial probability of remaining in remission for the 20 patients in CR at 3 months is 86% at 2 years. It is only 19% at 1 year for nine patients achieving a PR (Figure 2). The probability of disease free survival for patients in CR after MBE is 70% at 2 years as a consequence of late toxic deaths (Figure 3).

**Table II** Chemotherapy regimen and dose escalation (numbers of patients)

| *Melphalan* ($mg\,m^{-2}$) | *BCNU* ($mg\,m^{-2}$) 300 | 400 | 500 | 600 | *Etoposide* ($mg\,m^{-2}$) |
|---|---|---|---|---|---|
| 80 | 1 | | | | 1,200 |
| 100 | 1 | | | | 1,200 |
| 140 | 1 | 11 | 1 | 10 | 300 |
| 140 | | | 2 | 11 | 0 |

**Table III** Response to MBE

| | *No.* | *CR(%)* | *PR(%)* | *NR(%)* | *ED(%)* |
|---|---|---|---|---|---|
| Patients never in CR | 8 | 3 | 2 | | 3 |
| Patients in CR | 10 | 7 | | 1 | 2 |
| Patients relapsing | 20 | 10 | 7 | 2 | 1 |
| Overall | 38 | 20 (53) | 9 (24) | 3 (8) | 6 (16) |

**Figure 1** Overall probability of survival for 38 patients treated with MBE.

**Figure 2** Probability of remaining in remission for 20 patients in CR after MBE (——) and 9 patients in PR (– – –). The patient who remains in remission after entering PR on MBE received mantle radiotherapy to her residual disease which led to a CR.

**Figure 3** Probability of disease-free survival for 20 patients in CR after MBE (——) and 9 patients in PR (---). The patient who remains in remission after entering PR on MBE received mantle radiotherapy to her residual disease, which led to a CR.

*Bone marrow transplant*

Cryopreserved bone marrow was returned on day 0 according to standard procedures, patients receiving a median of $2.1 \times 10^8$ (range $1.42–6.0 \times 10^8$) frozen nucleated cells per kilogram of body weight. Median time to recover $1 \times 10^9 l^{-1}$ leucocytes was 25 days (range 9–115) and all patients achieved a satisfactory white cell graft. Median time to recover $50 \times 10^9 l^{-1}$ platelets was 51 days (range 17–314+) but four patients failed to reach this limit after 168, 253, 302 and 314 days. A level of $50 \times 10^9 l^{-1}$ platelets was reached as late as day 137 in one patient.

*Toxicity*

Acute toxicity included transient gastrointestinal side effects with nausea, vomiting, mucositis and diarrhoea. (See Table IV.) Clinical evidence of infections was common during the agranulocytic phase and required changes in wide spectrum antibiotics in 32 patients (84%). Transient renal toxicity measured by the serum creatinine level occurred in nine patients (24%). Transient changes in the liver function tests were noticed in 18 patients (47%) without clinical evidence of liver disease. Fatal cardiac toxicity occurred in one patient who died on day 26 of biventricular failure (patient 4, Table I). This patient had previously received extensive anthracycline therapy but autopsy did not reveal any evidence of specific drug-induced cardiac abnormalities.

The most severe toxicity of MBE was respiratory failure. There were seven toxic deaths from this cause, five occurring before 4 months and two late deaths. The five early deaths due to respiratory failure occurred on days 19, 58, 66, 78 and 103 and all of these five patients had received BCNU $600 \, mg \, m^{-2}$. Three of them had received previous bleomycin. The clinical course was acute pneumonitis leading to adult respiratory distress syndrome and autopsy showed interstitial pneumonitis compatible with drug-induced damage and no residual HD. The clinical pattern in the two patients with late respiratory failure was quite different. One patient who previously had chronic respiratory insufficiency probably related to bleomycin died on day 255 with respiratory failure of gradual onset (patient 3, Table I). He had received BCNU $300 \, mg \, m^{-2}$. No HD was found at autopsy and the lungs were fibrotic. Another patient (patient 29, Table I) died of respiratory failure on day 424. She had developed chronic pleural thickening with small lungs. A thoracotomy showed the lungs to be fibrotic but with no evidence of recurrent Hodgkin's disease. The pleura were resected but she died in the postoperative phase. Five additional patients had transient breathlessness with radiological evidence of pneumonitis following MBE but recovered fully. Among these five patients, two had received BCNU $600 \, mg \, m^{-2}$, one $500 \, mg \, m^{-2}$ and two $400 \, mg \, m^{-2}$. All patients with lung toxicity were treated with high dose steroids. Five (24%) of the 21 patients treated with $600 \, mg \, m^{-2}$ of BCNU died of early lung toxicity and two more had transient respiratory symptoms. Of the 17 patients treated with less than $600 \, mg \, m^{-2}$ of BCNU, none has died of early lung toxicity but four (24%) had non-fatal lung damage. The death rate is significantly different between patients who received $600 \, mg \, m^{-2}$ of BCNU and those who received lower doses of BCNU (Fisher's exact probability=0.04). The association between the dose of BCNU and the subsequent lung toxicity is very strong. We have analysed other possible contributory factors including previous chemotherapy and previous radiotherapy and we cannot demonstrate evidence implicating them in the lung damage. In particular, only one of the patients who died of heart or lung toxicity had ever received thoracic irradiation.

**Table IV** MBE toxicity according to WHO scale

| | *1* | *2* | *3* | *4* |
|---|---|---|---|---|
| Gastrointestinal | 8 | 16 | 10 | 3 |
| Infections | 4 | 11 | 16 | 1 |
| Renal | 4 | 3 | 1 | 1 |
| Hepatic | 7 | 6 | 4 | 1 |
| Pulmonary | 1 | 4 | 1 | 5 |
| Cardiac | 0 | 0 | 0 | 1 |

### Discussion

This analysis allows us to draw some conclusions about the use of melphalan and BCNU in high dose in combination for Hodgkin's disease and in particular to highlight our substantial concern about their toxicity to the lungs.

BCNU in high dose as a single agent is toxic to the lung but such toxicity is rare with doses below $800 \, mg \, m^{-2}$ (Perren *et al.*, 1987). Melphalan alone causes transient, clinically insignificant lung toxicity (Allen *et al.*, 1986). High dose melphalan, $200 \, mg \, m^{-2}$, did not produce significant lung toxicity during a prior study in a comparable group of patients (Russell *et al.*, 1988). With combination of doses of melphalan $140 \, mg \, m^{-2}$ and BCNU $600 \, mg \, m^{-2}$ we have seen a high rate of toxic deaths due to lung damage. This must represent an additive toxic effect and this incidence of severe toxicity precludes their usage together at these doses in these patients. The use of another alkylating agent, cyclophosphamide, given at high dose in combination with high dose BCNU and etoposide was not reported to cause such a high lung toxicity (Ahmed *et al.*, 1987; Carella *et al.*, 1987; Jagannath *et al.*, 1987; O'Reilly *et al.*, 1987; Philip *et al.*, 1986). In combination with high dose melphalan, $140 \, mg \, m^{-2}$, we now limit the dose of BCNU to a maximum of $500 \, mg \, m^{-2}$.

Nevertheless, there is no doubt that this approach leads to high remission rate for patients with very aggressive HD. In our study the overall response rate to MBE was 76%, with 53% of patients in CR at 3 months. The actuarial risk of relapse before 2 years is only 14% but median follow-up is only 1 year. The overall survival falls to 31% at 2 years as a consequence of the toxic deaths. Since the goal of these treatments is long-term disease-free survival, follow-up to 5 years is required to allow firm conclusions. The precise place of intensive chemotherapy for HD remains to be determined. To date all studies including this one have suffered from case selection and lack of a control arm. Studies are now required in relapsed or resistant patients to randomise conventional treatment against intensive regimens. Intensive treatments are likely to be associated with far less toxicity than hitherto, both because of what has been learned from studies of the type reported here and the relative lack of prior tissue damage in patients who receive high dose treatment earlier.

### References

AHMED, T., GINGRICH, S.A., CIAVARELLA, D. & 5 others (1987). High dose etoposide, cyclophosphamide +/− carmustine in conjunction with infusion of autologous bone marrow: a promising treatment for refractory or relapsed Hodgkin's disease. Proceedings of The Third International Conference on Malignant Lymphoma, Lugano, abstract, P17.

ALLEN, J., COOPER, D. JR, WHITE, D.A. & MATTHAY, R.A. (1986). Drug induced pulmonary disease. Part 1: cytotoxic drugs. *Am. Rev. Respir. Dis.*, **133**, 341.

CANELLOS, G.P. (1985). Bone marrow transplantation as salvage therapy in advanced Hodgkin's disease: allogenic or autologous. *J. Clin. Oncol.*, **3**, 1451.

CANELLOS, G., SELBY, P. & MCELWAIN, T.J. (1987). Chemotherapy for Hodgkin's disease. Section II: alternative combinations and new approaches. In *Hodgkin's Disease*, Selby, P. & McElwain, T.J. (eds) p. 285. Blackwell Scientific Publications: Oxford.

CARELLA, A.M., SANTINI, G., CONGIU, A. & 7 others (1987). Autologous bone marrow transplantation (ABMT) in the treatment of 25 advanced resistant Hodgkin's disease patients. Proceedings of The Third International Conference on Malignant Lymphoma, Lugano, abstract, P21.

FOX, K.A., LIPPMAN, S.M., CASSADY, J.R., HEUSINKVELD, R.S. & MILLER, T.P. (1987). Radiation therapy salvage of Hodgkin's disease following chemotherapy failure. *J. Clin. Oncol.*, **5**, 38.

HAGEMEISTER, F.B., TANNIR, N., MCLAUGHLIN, P. & 4 others (1987). MIME chemotherapy (Methyl-GAG, Ifosfamide, Methotrexate, Etoposide) as treatment for recurrent Hodgkin's disease. *J. Clin. Oncol.*, **5**, 556.

JAGANNATH, S., ARMITAGE, J.O., DICKE, K.A. & 8 others (1987). Autologous bone marrow transplantation (ABMT) for relapsed Hodgkin's disease. Proceedings of The Third International Conference on Malignant Lymphoma, Lugano, abstract, P18.

MCELWAIN, T.J. & POWLES, R.L. (1983). High dose intravenous melphalan for plasma cell leukemia and myeloma. *Lancet*, **ii**, 822.

MCELWAIN, T.J., PERREN, T.J. & SELBY, P.J. (1985). Etoposide containing combinations (OPEC, HOPE-Bleo) for advanced and recurrent Hodgkin's disease. Proceedings of the 14th International Congress of Chemotherapy, Kyoto, p. 29.

O'REILLY, S.E., CONNORS, J., VOSS, N. & 4 others (1987). Augmented cyclophosphamide, BCNU and etoposide (CBV) and autologous bone marrow transplantation (ABMT) in progressive Hodgkin's disease. Proceedings of The Third International Conference on Malignant Lymphoma, Lugano, abstract, P20.

PERREN, T.J., SELBY, P., MBIDDE, E. & 4 others (1987). High dose BCNU chemotherapy with autologous bone marrow transplantation and full dose radiotherapy for grade IV astrocytoma. *Bone Marrow Transplantation*, **2**, suppl. 1, 197.

PHILIP, T., DUMONT, J., TEILLET, F. & 7 others (1986). High dose chemotherapy and autologous bone marrow transplantation in refractory Hodgkin's disease. *Br. J. Cancer*, **53**, 737.

ROACH, M. III, KAPP, D.S., ROSENBERG, S.A. & HOPPE, R.T. (1987). Radiotherapy with curative intent: an option in selected patients relapsing after chemotherapy for advanced Hodgkin's disease. *J. Clin. Oncol.*, **5**, 550.

RUSSELL, J.A., SELBY, P., RUETHER, B.A. & 8 others (1989). High dose melphalan with autologous bone marrow transplantation in the treatment of relapsed or resistant Hodgkin's disease. *Bone Marrow Transplant.* (in the press).

SANTORO, A., VIVIANI, P., VALAGUSSA, P., BONFANTE, V. & BONADONNA, G. (1986). CCNU, Etoposide and Prednimustine (CEP) in refractory Hodgkin's disease. *Semin. Oncol.*, **13**, suppl. 1, 23.

SELBY, P. & MCELWAIN, T.J. (1987). *Hodgkin's Disease*. Blackwell Scientific Publications: Oxford.

TAYLOR, R.E., MCELWAIN, T.J., BARRETT, A. & PECKHAM, M.J. (1982). Etoposide as a single agent in advanced or relapsed lymphomas. A phase II study. *Cancer Chemother. Pharmacol.*, **7**, 175.

WORLD HEALTH ORGANIZATION (1979). *WHO Handbook for Reporting Results of Cancer Treatment*. WHO Offset Publication, p. 1. WHO: Geneva.